# The spinal notch signaling pathway plays a pivotal role in the development of neuropathic pain

**DOI:** 10.1186/1756-6606-5-23

**Published:** 2012-06-19

**Authors:** Xiao-Hua Liu, Nan Gu, Hai-Long Dong, Lize Xiong

**Affiliations:** 1 Department of Anesthesiology, Xijing Hospital, Fourth Military Medical University, Xi’an, Shaanxi, 710032, People's Republic of China

**Keywords:** Neuropathic pain, Notch signaling pathway, DAPT

## Abstract

**Background:**

The Notch signaling pathway has been shown to be involved in the development of the nervous system. Recent studies showed that Notch receptors and ligands are also expressed in the nervous system of adult animals. However, whether the Notch signaling pathway has a function in adults is not fully understood. The present study is designed to investigate the function of the Notch signaling pathway in nociceptive transmission, especially during neuropathic pain in adult rats.

**Results:**

We found that the Notch intracellular domain (NICD) is expressed in the DRG (Dorsal Root Ganglia), sciatic nerve and spinal cord in normal rats, and is upregulated in the sciatic nerve and spinal cord after spared nerve injury (SNI). Moreover, we used the γ-secretase (a key enzyme of the Notch signaling pathway) inhibitor DAPT to observe the effect of the Notch signaling pathway after SNI. We found that intrathecal DAPT significantly increased paw withdrawal thermal latency and mechanical threshold. Mechanical hyperalgesia occurring after SNI could be significantly reversed by DAPT in a dose-dependent manner.

**Conclusions:**

These results suggest that the Notch signaling pathway participates in the induction and maintenance of neuropathic pain, which indicates that the Notch pathway maybe a potential drug target for neuropathic pain treatment.

## Background

Chronic pain is a major health problem. Therefore, providing better treatment and better methods for preventing chronic pain is of clinical importance. In particular neuropathic pain, which has a higher morbidity, is severely debilitating, and is largely resistant to treatments. However, the underlying mechanisms of neuropathic pain are still unknown. Several mechanisms have been shown to be involved in the initiation and propagation of neuropathic pain, including increased excitability and reduced thresholds of primary sensory neurons, altered spinal cord synaptic processing, loss of inhibitory interneurons and modifications of brain stem input to the spinal cord [1-3].

The Notch signaling pathway is a highly conserved pathway in evolution, which is involved in many physiological and pathological processes, such as vasculogenesis, immunological processes, development, learning and memory, and tumor formation [4-7]. Notch is one of the cell-surface receptors that regulate cell-fate decision in the developing nervous system. Ligands such as Delta and Jagged bind to Notch receptors, resulting in proteolytic cleavage of Notch into two sections: an extracellular domain and a transmembrane domain. The latter cleavage is completed by the γ-secretase enzyme and ADAM, resulting in the release of a Notch intracellular domain (NICD) that translocates to the nucleus, where it regulates transcription [8]. Some findings suggest that activation of Notch signaling can contribute to neuronal death, generation and activation of microglial cells and astrocytes, inhibition of neurite growth [9-11], more dendritic branching [12], differentiation of oligodendrocyte progenitors and demyelination in both the peripheral nervous system [13] and the CNS (central nervous system) [14].

Recent studies have demonstrated that activation of the Notch signaling pathway potentiates synaptic transmission in the hippocampus [15]. The role of the Notch signaling pathway in sensory sensation, particularly in nociceptive transmission remains unclear. Therefore, this study observed the effect of the Notch signaling pathway on nociceptive responses induced by nerve injury.

## Results and discussion

**Figure 1:**
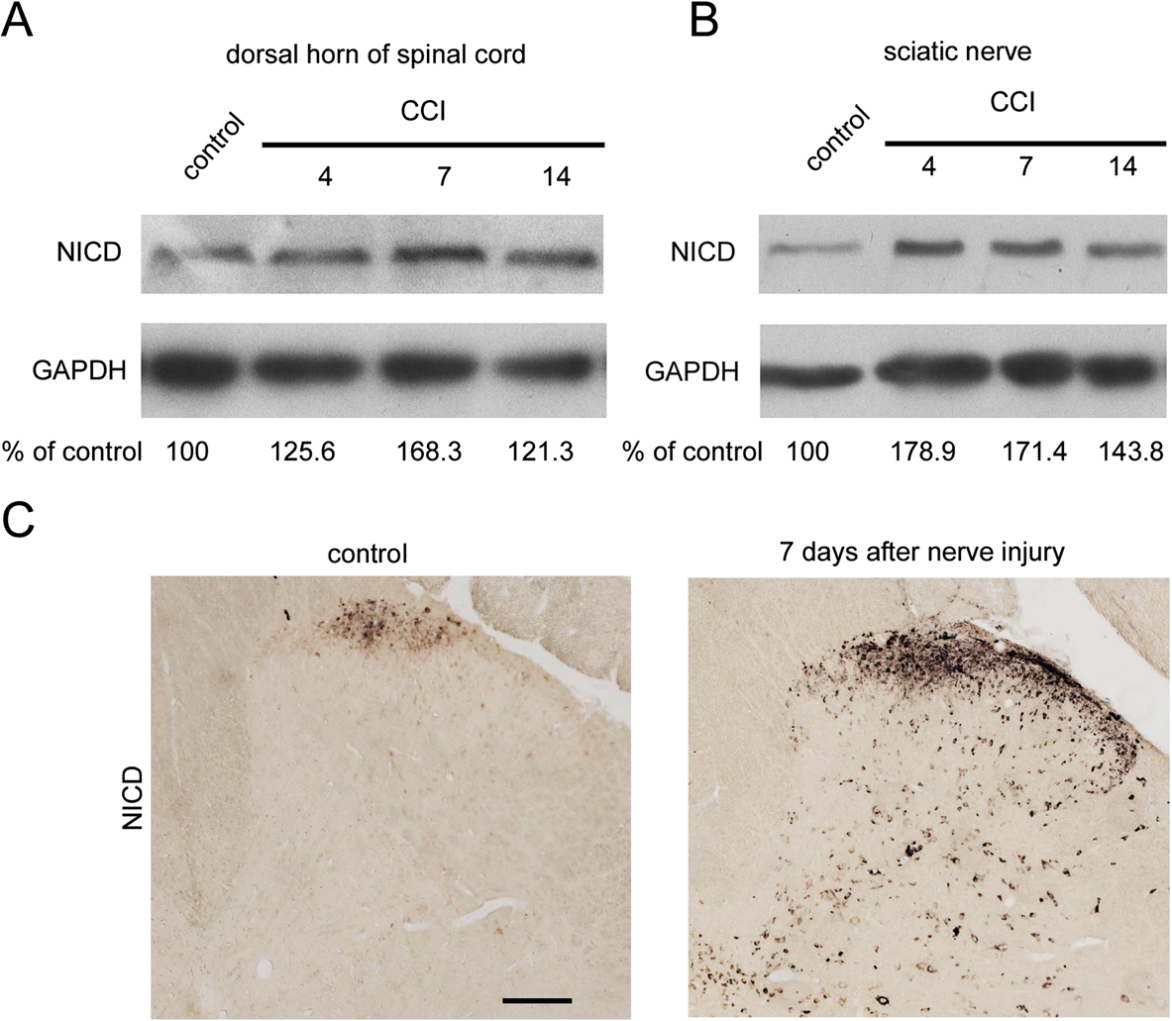
** Change in expression of NICD in the sciatic nerve and dorsal horn of the spinal cord in control and CCI model rats.** (**A**) Immunoblotting showed the expression of NICD in the dorsal horn of the spinal cord. NICD increased after nerve injury (CCI model) (n = 3). (**B**) Immunoblotting showing the expression of NICD in the sciatic nerve. NICD increased after nerve injury (CCI model) (n = 3). (**C**) Immunostaining showed that the expression of NICD was significantly increased after nerve injury (CCI model). Scale bar, 100 μm.

**Figure 2:**
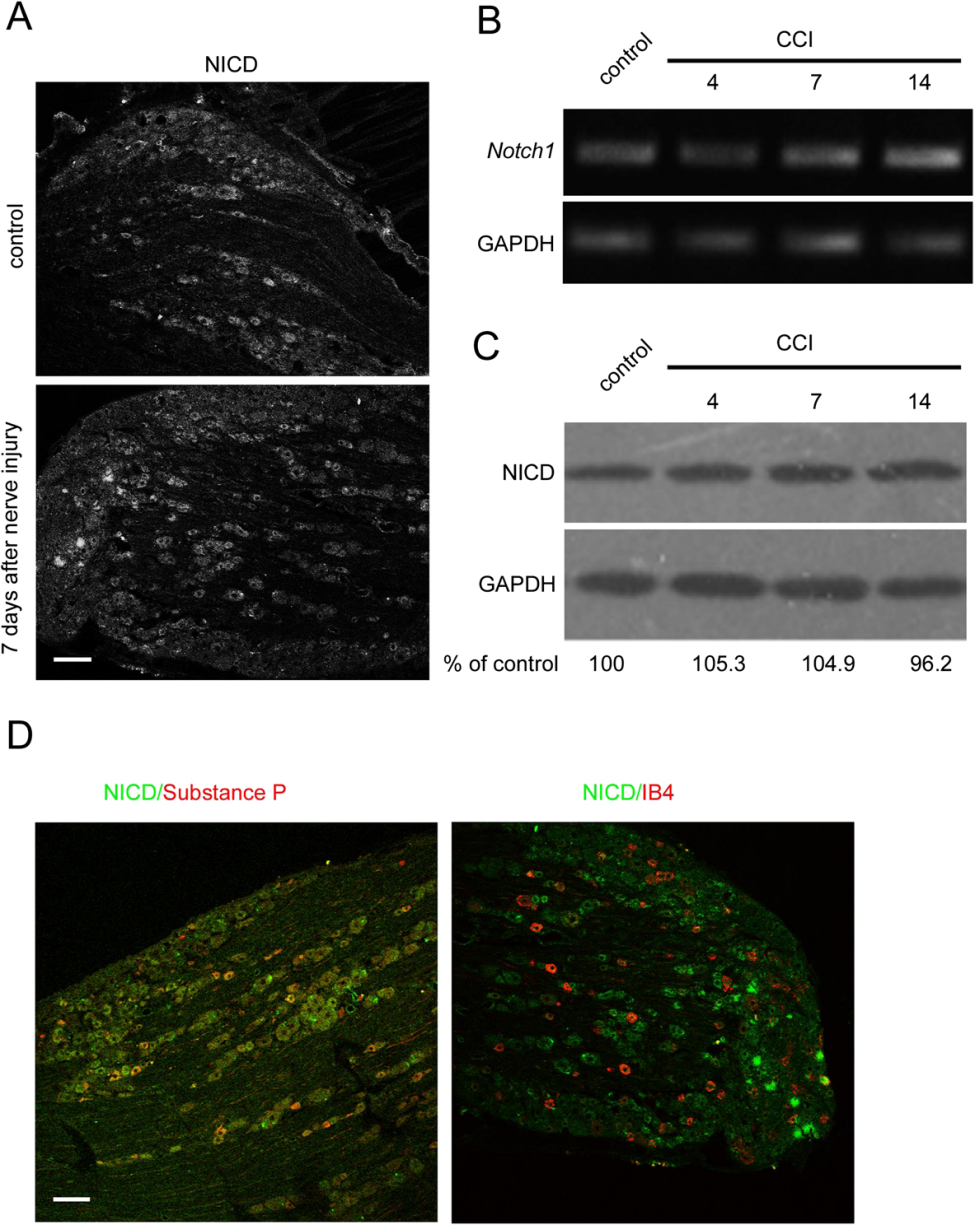
** The change in expression of NICD in DRG neurons in control and CCI model rats.** (**A**) Immunostaining showing the expression of NICD in the DRG. NICD was present in small neurons in the DRG, and nerve injury did not induce significant change. Scale bar, 100 μm. (**B**) RT-PCR revealed Notch1 expression in DRG tissue, while nerve injury did not induce a significant change. (**C**) Immunoblotting showed that in the rat DRG, the levels of NICD did not markedly change after nerve injury (CCI model) (n = 3). The data represents three independent experiments. (**D**) Immunostaining revealed that NICD co-localized with substance P, but did not co-localize with IB4 in the DRG. Scale bar, 100 μm.

Our present study shows that NICD expression in the rat sciatic nerve and dorsal horn of the spinal cord is markedly increased following peripheral nerve injury. Seven days after induction of the unilateral CCI (Sciatic Nerve Chronic Constriction Injury Model) model, immunoblot analyses showed that NICD protein levels in the dorsal horn of the spinal cord increased to ~168.3% of the control (Figure 1A). NICD protein levels in the sciatic nerve increased to ~171.4% of the control (Figure 1B), and the density of the NICD-immunoreactive product in the spinal cord was increased significantly in laminas I-II at 7 days after nerve injury in the CCI model (Figure 1C). However, both NICD mRNA and protein levels in rat DRG neurons were not altered significantly following peripheral nerve injury (Figure 2A-C). Co-immunostaining of NICD and Substance P, or IB4, indicated that NICD partially co-localized with peptidergic small DRG neurons (Figure 2D).

**Figure 3:**
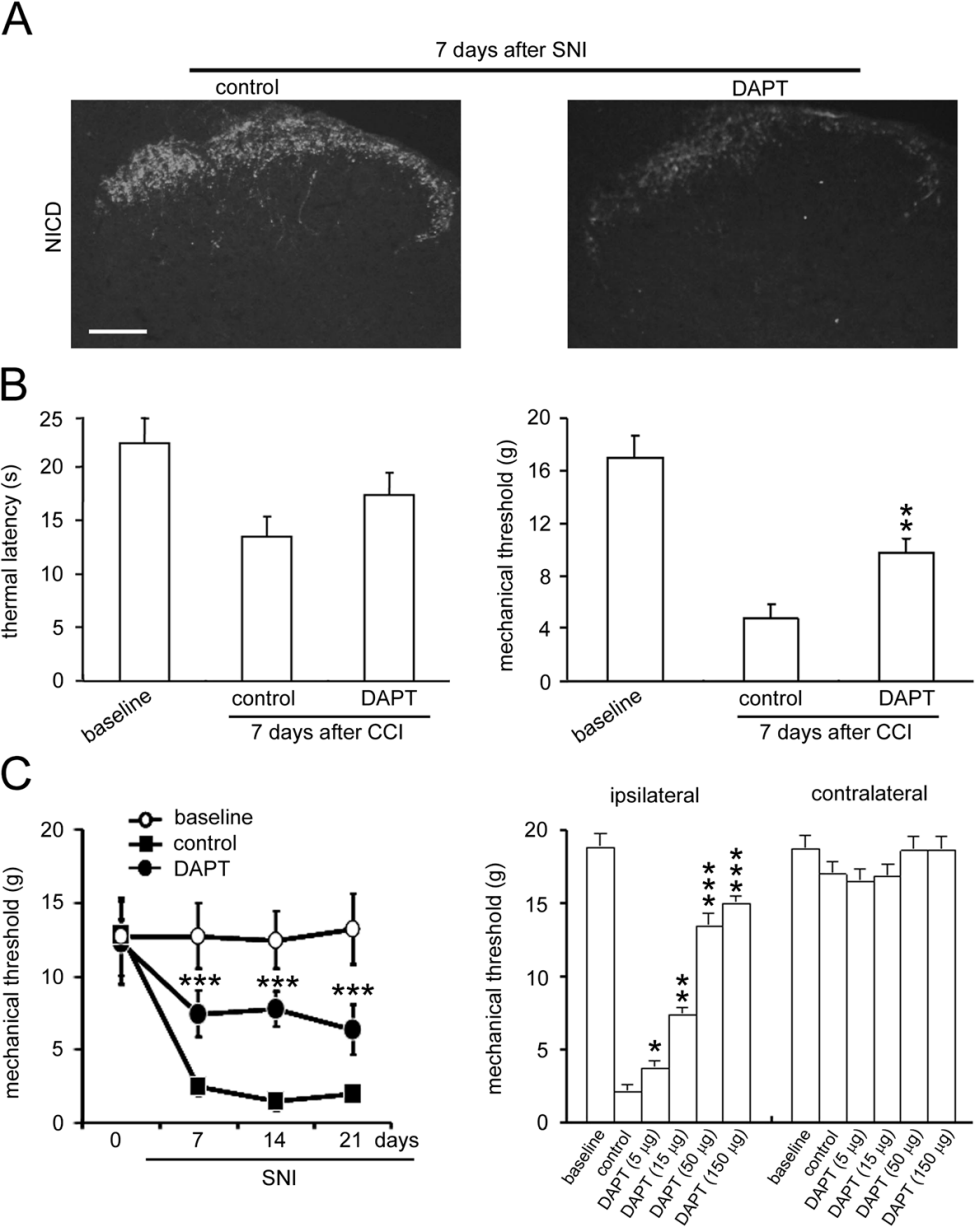
** DAPT inhibits thermal hyperalgesia and mechanical allodynia induced by CCI and SNI.** (**A**) Immunostaining revealed that the increase of NICD expression in the spinal cord after nerve injury was significantly inhibited by injection with DAPT. Scale bar, 100 μm. (**B**) Intrathecal (i.t.) administration of DAPT inhibits thermal hyperalgesia and mechanical allodynia 7 days after CCI. (n = 10). (**C**) Intrathecal (i.t.) DAPT inhibits mechanical allodynia after SNI in a dose-dependent manner. * *P* < 0.05, ** *P* < 0.01, *** *P* < 0.01 versus SNI rats treated with vehicle (mean ± s.e.m., two-way ANOVA with a *post hoc* Bonferroni’s test, n = 15).

To test whether nerve injury-induced NICD expression contributes to pain hypersensitivity, DAPT, a γ-secretase enzyme inhibitor, was injected intrathecally (i.t.) before nerve injury. Spinal NICD expression levels decreased significantly 7 days after in the SNI model(P < 0.05) (Figure 3A). We found that DAPT suppressed thermal hyperalgesia and mechanical allodynia induced by CCI (Figure 3B) and inhibited the mechanical allodynia induced by the SNI model in a dose-dependent manner (Figure 3C). Thus, the marked increase of NICD in the dorsal spinal cord could be an underlying mechanism for the development of neuropathic pain.

In this study, we found that NICD was upregulated in the sciatic nerve and spinal cord after nerve injury. DAPT, an inhibitor of a key enzyme in the Notch signaling pathway, reversed the thermal hyperalgesia and mechanical allodynia induced by nerve injury. We therefore speculate that the Notch signaling pathway is involved in the development and maintenance of neuropathic pain.

Recently, a number of studies have shown that Notch participates in synaptic plasticity, learning and memory in rodents [16-18], long-term memory formation in Drosophila [19-21], and locomotive behavior in C. elegans [22]. Decline of Notch1 expression by 50% (using an anti-sense strategy) resulted in reduced longterm potentiation (LTP) [18].

In our present study, we demonstrated that NICD is expressed in the DRG, sciatic nerve and spinal cord in adult rats, and that NICD was increased in the sciatic nerve and spinal cord after nerve injury, indicating its potential role in the development of neuropathic pain. On the other hand, although the change of NICD expression in the DRG was not statistically significant compared with the sciatic nerve and spinal cord, there was an increasing trend. Our results showed that expression of NICD in DRG neurons was upregulated from ~53% to ~61%, while western-blot results showed no apparent change in NICD protein levels after nerve injury. Therefore, we believe that NICD may be transported to the central terminals after production, so that the expression of NICD is upregulated in the spinal cord and sciatic nerve. Consistent with our results, some studies have shown that the Notch signaling pathway enhanced calcium influx in DRG neurons and also potentiated synaptic transmission in the hippocampus [15,23]. In addition, in the spinal cord compression injury model, the expression of Notch1 increased after spinal cord injury [24]. These results indicated that the Notch signaling pathway is involved in nociceptive transmission in the spinal cord.

## Conclusion

In conclusion, activation of the Notch signaling pathway may contribute to the generation and development of neuropathic pain. The upregulation of NICD in the spinal cord after nerve injury is an important factor for the development of neuropathic pain, and inhibition of γ-secretase may be a potential therapeutic strategy for treating neuropathic pain.

## Materials and methods

### Animals

Adult male Sprague–Dawley rats (Laboratory Animal Center of Fourth Military Medical University (FMMU), China) weighing 180–220 g, were used in the study. The animals were kept in plastic boxes at 22-26°C with free access to food and water. All rats were allowed to acclimatize to the housing facilities and were handled daily for at least 3 days. All experimental protocols and animal handling procedures were approved by the FMMU Animal Care and Use Committee and adhered to the guidelines for the ethical treatment of animals established by the International Association for the Study of Pain.

### RT-PCR

Notch1 was identified in the rat DRG by RT-PCR using the following primers: 5’- TGAGATGCTCCCAGCCAAGT 3’ and 5’- AGATGTATGAAGACTCAAAGGG -3’(5 min at 95°C, 30s at 95°C, 30s at 58°C, 30s at 68°C; 40cycle).

### Immunoblotting

The DRG, sciatic nerve and spinal cord tissues were sonicated/homogenized and centrifuged. Thirty micro-grams of protein was extracted from the above preparations, and loaded and separated on a 10% (w/v) SDS-polyacrylamide gel. After transfer, blots were incubated overnight at 4°C with separately with NICD (1:1000; Abcam, London, UK) and GAPDH (1:10000; Chemicon, Temecula, CA, USA) primary antibodies. Blots were visualized using enhanced chemiluminescence (ECL; Roche, Basel, Switzerland). Quantification was based on 3 independent experiments. The ECL signal intensity of NICD versus GADPH was quantified using the NIH image program.

### Immunostaining

Adult male rats were fixed with 4% (w/v) paraformaldehyde. For all experimental groups, 12 μm-thick sections of the fixed L4-5 DRG and L4-5 spinal cord segments were cut. The sections were processed with indirect immunofluorescent histochemistry. Antisera were diluted in phosphate buffered saline (PBS) containing 0.3% (v/v) Triton X-100 and 1% (w/v) bovine serum albumin. Briefly, the sections were incubated with a mixture of rabbit anti-NICD antibodies (1:500) overnight at 4°C. The sections were rinsed three times in PBS and then incubated with fluorescence-conjugated donkey anti–rabbit (1:100) and rhodamine-conjugated donkey anti-goat IgG (1:100; Jackson Immuno Research, West Grove, PA, USA) for 30 min at 37°C. After several rinses in PBS, the sections were placed under a Leica SP2 confocal microscope (Leica Microsystem, Heidelberg, Germany) and images were captured.

### SNI model of neuropathic pain

The left SNI model was performed as previously described [25]. Briefly,Under pentobarbital sodium anesthesia (40 mg/kg), an incision was made on the left side skin of the hind leg. The muscle and strands of the bicep femoris were separated to expose the sciatic nerve-trunk and carefully separate its three branches: tibial, common peroneal and sural nerves. After separation, double ligation of the tibial and common peroneal was performed using a 5.0 silk thread, and 2–3 mm of the nerves distal to the ligation were removed, taking care to avoid inducing any lesion, or stretching the intact sural nerve. The muscle and skin incisions were then closed separately. Sham surgery groups only underwent sciatic nerve-trunk exposure and exposure of their branches, but did not undergo ligation and transection. A skin incision was made to blind the observer during behavioral testing. Animals were habituated to the tester, the environment, and to the handling procedures before commencement of testing.

### CCI model of neuropathic pain

CCI of the sciatic nerve was used as a model of neuropathic pain, and was adapted to the mouse [26]. Briefly, under pentobarbital sodium anesthesia (40 mg/kg, intra-peritoneal injection), the left sciatic nerve was exposed by blunt dissection around the nerve proximal to the trifurcation and loosely ligated at four sites using a 4-0 chromic catgut suture (Jinhuan Medical Instruments Co., Ltd., Shanghai, China). The distance between the two adjacent ligatures was 1 mm. The wound was irrigated with saline (0.9% (w/v)) and closed in two layers with 4-0 silk (facial plane) and surgical skin staples. For the sham operation, the sciatic nerve was exposed but not ligated.

### Intrathecal surgery and drug injection

The permanent intrathecal catheter (PE-10 polyethylene tube) was inserted through the gap between the T3 and T4 vertebrae and extended slowly to the subarachnoid space of the lumbar enlargement (L4 and L5 segments) under sodium pentobarbital (40 mg/kg, intraperitoneal (i.p.) anesthesia. The catheter was filled with sterile NS(Normal Saline) (approximately 4 μL), and the outer end was plugged in the skin to avoid the paws. The cannulated rats were allowed to recover for 3-4 days. Excluding rats with any neurological deficits, at the end of each experiment, pontamine sky blue was injected via the i.t. catheter to prove its effectiveness. DAPT (Sigma, St. Louis, MO, USA), a potent inhibitor of Notch signaling pathway activation, was freshly dissolved daily in dimethy sulphoxide (DMSO) to a concentration of 50 mM. Drug or vehicle (DMSO) was injected over a period of 1 min via the catheter at a volume of 10 μL, followed by 5 μL NS for flushing, and all drugs were injected once 30 min before surgery.

### Behavioral testing

Animals were housed in plastic boxes and allowed to acclimatize for at least 24 h before testing commenced. This allowed animals to become familiarized with the handling and environment. Baseline assessments of nociceptive sensitivity were taken for all animals 24 h before surgery. All behavioral pain tests were conducted at the same time each day, between 10.00 AM and 1.00 PM.

### Thermal hyperalgesia testing

Animals were placed in a clear glass cage with a glass floor and allowed to acclimatize for 30 min. For the once pre- and post-drug experiment, a beam of radiant heat was applied to the middle of the plantar surface of the operation-lateral paws on days 7, 14, and 21 after drug or vehicle administration. Withdrawal latency was recorded. Radiant heat was adjusted so that the baseline latency was 20- 25 s. The cutoff time was 30 s. The latency was examined at least three times, with at least 10 min between the two consecutive stimuli on the same paw.

### Mechanical allodynia testing

For the once pre- and post-drug experiment, the von Frey monofilament was conducted on days 7, 14, and 21 after drug or vehicle administration. The degree of hyperalgesia was expressed as the difference between the probability of the hyperalgesia response of the right and left paw for each animal.

### Data analysis and statistics

All numerical data are presented as the mean ± S.E.M. Statistical significance was determined as P < 0.05 using the student’s paired *t*-test and n refers to the number of neurons studied.

## Abbreviations

ADAM, A Disintegrin and A Metalloprotease; CNS, Central Nervous System; CCI, Sciatic Nerve Chronic Constriction Injury Model; DRG, Dorsal Root Ganglia; DAPT, N-[N-(3,5-difluorophenacetyl)-L-alanyl]-S-phenylglycine t-butyl ester; GAPDH, Glyceraldehyde-3-phosphate dehydrogenase; MWT, Mechanical Withdrawal Threshold; NICD, Notch intracellular domain Notch; NS, Normal Saline; SNI, Spared Nerve Injury Model.

## Competing interests

The authors declare that they have no competing interests.

## Authors’ contributions

YYS, LL and LZX conceived and designed the study. YYS, LL, XHL, NG and HLD performed the experiments. All authors read and approved the final manuscript.

## Authors’ information

Yan-Yan Sun and Li Li are the first co authors.

## References

[B1] Woolf CJ Central sensitization: implications for the diagnosis and treatment of pain Pain 2011 152 S2 S15 10.1016/j.pain.2010.09.030 20961685 PMC3268359

[B2] Costigan M Scholz J Woolf CJ Neuropathic pain: a maladaptive response of the nervous system to damage Annu Rev Neurosci 2009 32 1 32 10.1146/annurev.neuro.051508.135531 19400724 PMC2768555

[B3] Ji RR Woolf CJ Neuronal plasticity and signal transduction in nociceptive neurons: implications for the initiation and maintenance of pathological pain Neurobiol Dis 2001 8 1 10 10.1006/nbdi.2000.0360 11162235

[B4] Louvi A Artavanis-Tsakonas S Notch signalling in vertebrate neural development Nat Rev Neurosci 2006 7 93 102 10.1038/nrn1847 16429119

[B5] Swift MR Weinstein BM Arterial-venous specification during development Circ Res 2009 104 576 588 10.1161/CIRCRESAHA.108.188805 19286613

[B6] Yuan JS Kousis PC Suliman S Visan I Guidos CJ Functions of notch signaling in the immune system: consensus and controversies Annu Rev Immunol 2010 28 343 365 10.1146/annurev.immunol.021908.132719 20192807

[B7] Lathia JD Mattson MP Cheng A Notch: from neural development to neurological disorders J Neurochem 2008 107 1471 1481 10.1111/j.1471-4159.2008.05715.x 19094054 PMC4544712

[B8] Pierfelice T Alberi L Gaiano N Notch in the vertebrate nervous system: an old dog with new tricks Neuron 2011 69 840 855 10.1016/j.neuron.2011.02.031 21382546

[B9] Wines-Samuelson M Shen J Presenilins in the developing, adult, and aging cerebral cortex Neuroscientist 2005 11 441 451 10.1177/1073858405278922 16151045

[B10] Chenn A A top-NOTCH way to make astrocytes Dev Cell 2009 16 158 159 10.1016/j.devcel.2009.01.019 19217415

[B11] Ferrari-Toninelli G Bonini SA Bettinsoli P Uberti D Memo M Microtubule stabilizing effect of notch activation in primary cortical neurons Neuroscience 2008 154 946 952 10.1016/j.neuroscience.2008.04.025 18495362

[B12] Brandt MD Maass A Kempermann G Storch A Physical exercise increases Notch activity, proliferation and cell cycle exit of type-3 progenitor cells in adult hippocampal neurogenesis Eur J Neurosci 2010 32 1256 1264 10.1111/j.1460-9568.2010.07410.x 20950279

[B13] Morrison SJ Perez SE Qiao Z Verdi JM Hicks C Weinmaster G Anderson DJ Transient Notch activation initiates an irreversible switch from neurogenesis to gliogenesis by neural crest stem cells Cell 2000 101 499 510 10.1016/S0092-8674(00)80860-0 10850492

[B14] Tanigaki K Nogaki F Takahashi J Tashiro K Kurooka H Honjo T Notch1 and Notch3 instructively restrict bFGF-responsive multipotent neural progenitor cells to an astroglial fate Neuron 2001 29 45 55 10.1016/S0896-6273(01)00179-9 11182080

[B15] Alberi L Liu S Wang Y Badie R Smith-Hicks C Wu J Pierfelice TJ Abazyan B Mattson MP Kuhl D Pletnikov M Worley PF Gaiano N Activity-induced Notch signaling in neurons requires Arc/Arg3.1 and is essential for synaptic plasticity in hippocampal networks Neuron 2011 69 437 444 10.1016/j.neuron.2011.01.004 21315255 PMC3056341

[B16] Costa RM Honjo T Silva AJ Learning and memory deficits in Notch mutant mice Curr Biol 2003 13 1348 1354 10.1016/S0960-9822(03)00492-5 12906797

[B17] Saura CA Choi SY Beglopoulos V Malkani S Zhang D Shankaranarayana Rao BS Chattarji S Kelleher RJ 3rd Kandel ER Duff K Kirkwood A Shen J Loss of presenilin function causes impairments of memory and synaptic plasticity followed by age-dependent neurodegeneration Neuron 2004 42 23 36 10.1016/S0896-6273(04)00182-5 15066262

[B18] Wang Y Chan SL Miele L Yao PJ Mackes J Ingram DK Mattson MP Furukawa K Involvement of Notch signaling in hippocampal synaptic plasticity Proc Natl Acad Sci U S A 2004 101 9458 9462 10.1073/pnas.0308126101 15190179 PMC438998

[B19] Ge X Hannan F Xie Z Feng C Tully T Zhou H Xie Z Zhong Y Notch signaling in Drosophila long-term memory formation Proc Natl Acad Sci U S A 2004 101 10172 10176 10.1073/pnas.0403497101 15220476 PMC454384

[B20] Matsuno M Horiuchi J Tully T Saitoe M The Drosophila cell adhesion molecule klingon is required for long-term memory formation and is regulated by Notch Proc Natl Acad Sci U S A 2009 106 310 315 10.1073/pnas.0807665106 19104051 PMC2606903

[B21] Presente A Boyles RS Serway CN de Belle JS Andres AJ Notch is required for long-term memory in Drosophila Proc Natl Acad Sci U S A 2004 101 1764 1768 10.1073/pnas.0308259100 14752200 PMC341850

[B22] Chao MY Larkins-Ford J Tucey TM Hart AC lin-12 Notch functions in the adult nervous system of C. elegans BMC Neurosci 2005 6 45 10.1186/1471-2202-6-45 16011804 PMC1181819

[B23] Shideman CR Reinardy JL Thayer SA gamma-Secretase activity modulates store-operated Ca2+ entry into rat sensory neurons Neurosci Lett 2009 451 124 128 10.1016/j.neulet.2008.12.031 19114088 PMC2634821

[B24] Chen J Leong SY Schachner M Differential expression of cell fate determinants in neurons and glial cells of adult mouse spinal cord after compression injury Eur J Neurosci 2005 22 1895 1906 10.1111/j.1460-9568.2005.04348.x 16262629

[B25] Decosterd I Woolf CJ Spared nerve injury: an animal model of persistent peripheral neuropathic pain Pain 2000 87 149 158 10.1016/S0304-3959(00)00276-1 10924808

[B26] Bennett GJ Xie YK A peripheral mononeuropathy in rat that produces disorders of pain sensation like those seen in man Pain 1988 33 87 107 10.1016/0304-3959(88)90209-6 2837713

